# Secondary obsessive-compulsive syndromes: a systematic literature review resulting in 228 suspected cases

**DOI:** 10.1038/s41380-025-03395-1

**Published:** 2025-12-23

**Authors:** Kimon Runge, Bernd Feige, Miriam A. Schiele, Katharina von Zedtwitz, Alexander Maier, Nils Stöcker, Raphael J. Dressle, Juan C. Baldermann, Simon J. Maier, Kathrin Nickel, Harald Prüss, Volker A. Coenen, Ulrich Voderholzer, Katharina Domschke, Ludger Tebartz van Elst, Dominique Endres

**Affiliations:** 1 Department of Psychiatry and Psychotherapy, Medical Center - University of Freiburg, Faculty of Medicine, University of Freiburg, Freiburg, Germany; 2 Department of Neurology, Faculty of Medicine, University of Cologne, Cologne, Germany; 3 Department of Psychiatry and Psychotherapy, Faculty of Medicine and University Hospital Cologne, University of Cologne, Cologne, Germany; 4 Department of Neurology and Experimental Neurology, Charité - Universitätsmedizin Berlin, Berlin, Germany; 5 German Center for Neurodegenerative Diseases (DZNE) Berlin, Berlin, Germany; 6 Department of Stereotactic and Functional Neurosurgery, Medical Center - University of Freiburg, Faculty of Medicine, University of Freiburg, Freiburg, Germany; 7 Center for Deep Brain Stimulation, University of Freiburg, Freiburg, Germany; 8 Schoen Clinic Roseneck, Prien am Chiemsee, Germany; 9 Department of Psychiatry and Psychotherapy, University Hospital Munich, Munich, Germany; 10 German Center for Mental Health (DZPG), Partner Site Berlin/Potsdam, Berlin, Germany

**Keywords:** Diagnostic markers, Neuroscience

## Abstract

Secondary forms of obsessive-compulsive disorder (OCD) have clear underlying organic causes and are recognized as distinct nosological entities in the latest international classification systems. This study aims to provide a systematic overview of published cases of suspected secondary obsessive-compulsive syndromes. A systematic literature search of PubMed, Embase, Web of Science, and PsycINFO was conducted oriented on PRISMA criteria. Cases from case studies/series of patients with suspected secondary obsessive-compulsive syndromes and/or secondary obsessive-compulsive symptoms were included. Cases of obsessive-compulsive symptoms due to pediatric acute-onset neuropsychiatric syndrome (PANS) and pediatric autoimmune neuropsychiatric disorders associated with streptococcal infections (PANDAS) were excluded. Overall, 228 cases of suspected secondary obsessive-compulsive syndromes were identified from 189 publications. Causal factors included brain lesions (25.4%), genetic syndromes (24.1%), head trauma (12.3%), autoimmune-inflammatory processes (11.8%), tumors (8.3%), neurodegeneration (7.5%), seizures (4.8%), pathogens (3.9%), metabolic processes (1.3%), or other reasons (0.4%). The age of the affected patients varied considerably (mean 37.3 ± 21.2 years, range 4–94 years, n = 226). Diagnostic abnormalities were identified through brain imaging (magnetic resonance imaging/computer tomography) in 66.2% of the sample and via blood analysis in 23.9%. In cases reporting the regions of the brain involvement, frontal lobe (34.3%) and the basal ganglia (26.5%) were mostly affected. The findings highlight a variety of suspected causes of secondary obsessive-compulsive syndromes, most frequently brain lesions, genetic syndromes, head trauma, and autoimmune-inflammatory processes. Identifying secondary obsessive-compulsive symptoms informed personalized therapies in a subgroup of published cases.

## Introduction

Obsessive-compulsive disorder (OCD) is among the most common mental disorders, with lifetime prevalence rates ranging between 2–3% and is often associated with a significant reduction in quality of life [1–3]. In contrast to primary forms of OCD, secondary forms of obsessive-compulsive syndromes can be attributed to clear organic causes, such as brain lesions in strategic regions (e.g., after strokes) or inflammatory brain changes (e.g., caused by autoimmune processes) [4]. Secondary obsessive-compulsive syndromes are recognized as distinct nosological entities in the latest versions of the International Statistical Classification of Diseases and Related Health Problems 11^th^ edition (ICD-11, coded as 6E64) [5] and the 5^th^ version of the Diagnostic and Statistical Manual of Mental Disorders (DSM-5, coded as 294.8) [6]. However, knowledge about the prevalence, spectrum of causes, and optimal diagnostic approach for the detection of secondary obsessive-compulsive syndromes still need to be investigated. Recommendations for diagnostic assessments are therefore limited in current international OCD guidelines (see Table 1) [7–9]. However, autoimmune OCD forms, for example, paradigmatically show that detection of secondary OCD might influence therapeutic approaches in some patients [10–12]. The detection of secondary forms and the resulting personalized treatment approaches could ideally lead to a relevant reduction in high treatment-resistant rates in patients with OCD [13, 14]. Therefore, there is an urgent need for research on secondary obsessive-compulsive syndromes and secondary obsessive-compulsive symptoms (OCS).

**Table 1:** Recommended diagnostic assessment for OCD patients according to different international guidelines.

Diagnostic Assessment	Guideline of the American Psychiatric Association (2007 with update 2013)	NICE Guideline (2005)	DGPPN S3-Guideline (2022)
**Screening-Questions**	Recommended	Recommended	Recommended
**Check with diagnostic criteria**	Recommended (DSM-IV)	N/A	Recommended (ICD-10, DSM-5, ICD-11)
**Rating the severity of OCD**	Recommended (e.g., Y-BOCS)	N/A	Recommended (e.g., Y-BOCS)
**Follow-up assessment**	Recommended (e.g., Y-BOCS)	N/A	Recommended (e.g., Y-BOCS)
**Evaluate the safety of the patient and others**	Recommended	Recommended	N/A
**Evaluation of well-being, functioning, and quality of life**	Recommended	Recommended	Recommended
**Somatic differential diagnostics**	Assessment of general medical history (current general medical conditions, recent or relevant hospitalizations, and any history of head trauma, loss of consciousness, or seizures)	N/A	All OCD patients should undergo somatic and neurological examination at initial presentation to rule out somatic disease. In patients with late-onset OCD ( > 50 years), a neuropsychological screening examination and structural imaging (CT or better MRI) of the brain should also be performed

*CT*, computer tomography, *DSM*, diagnostic and statistical manual of mental disorders, *ICD* international statistical classification of diseases and related health problems, *MRI* magnetic resonance imaging, *N/A* not available, *OCD* obsessive compulsive disorder, *Y-BOCS* yale-brown obsessive compulsive scale.

**The rationale of this study** was to provide the first systematic overview of suspected cases of secondary obsessive-compulsive syndromes from the literature. For this purpose, all published cases were summarized from case reports and series available in relevant medical databases. Particular attention was paid to patient characteristics, diagnostic findings, and treatment experiences.

## Material and methods

### Eligibility criteria

Original research findings comprising case reports and case series reporting individual patient data on secondary obsessive-compulsive syndromes or secondary OCS were included. Cross-sectional studies with no individual patient data as well as articles published in languages other than English, German, or Spanish were excluded. Patients of all age groups were included. For patients with comorbid mental illness, only cases with leading and/or severe OCS were included. Reports were also included if they were published as abstracts, poster presentations, letters to the editor or other correspondences. They were excluded if an assessment of the inclusion criteria was not possible based on the available information. Case reports on primary OCD, with improvement of OCS by organic factors (e.g., clinical improvement as a result of new brain lesions), with pediatric acute-onset neuropsychiatric syndrome (PANS), and pediatric autoimmune neuropsychiatric disorders associated with streptococcal infections (PANDAS) were excluded given that the association between PANS/PANDAS and OCS is already well established [15, 16]. Following ICD-11 exclusion criteria, also substance-induced cases, tic-disorders and cases with delirium were excluded [5].

### Literature search

The literature search was performed in the PubMed, Embase, Web of Science, and PsycINFO databases oriented on PRISMA criteria [17]. The literature search was performed on May 2, 2022 applying the following search term without restriction of publication date: ((OCD OR OCS OR obsessive-compulsive) AND (organic OR autoimmune OR encephalitis OR PANDAS OR PANS OR genetic OR syndromal OR tumor OR malign* OR seizure OR encephalopathy OR neurodegeneration OR degenerative OR basal ganglia OR frontal brain OR dementia OR trauma OR stroke OR lesion OR intoxication) AND (case report OR case study OR case series)) NOT (osteochondritis OR osteochondrosis OR osteofascial compartment syndrome OR orbital compartment syndrome OR oral contraceptive OR outerbridge cartilage score OR occipital condyle syndrome OR ovarian carcinosarcoma).

**Fig. 1:**
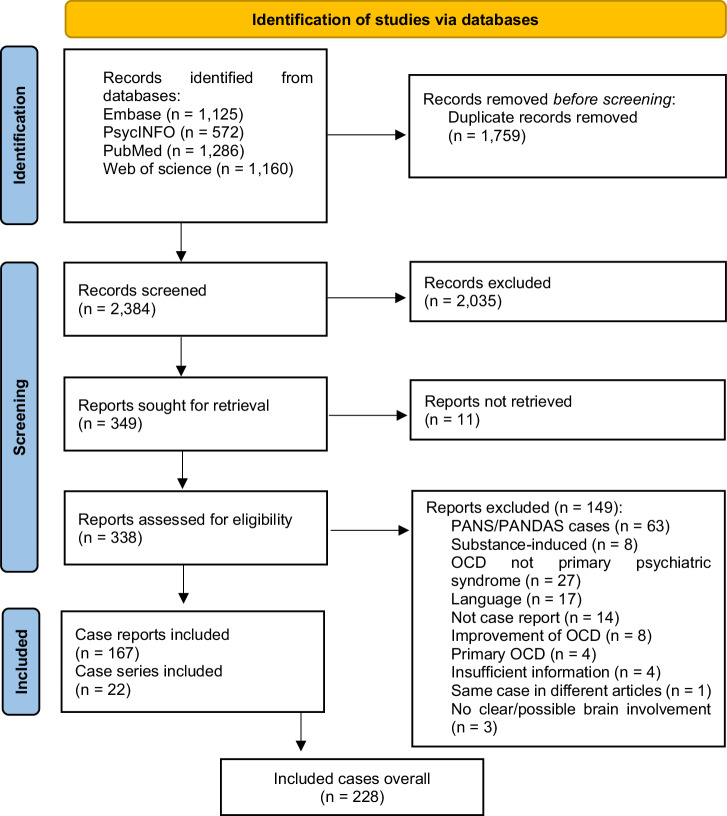
PRISMA flow diagram for literature search [17]. OCD, obsessive-compulsive disorder; PANDAS, pediatric autoimmune neuropsychiatric disorders associated with streptococcal infections; PANS, pediatric acute-onset neuropsychiatric syndrome.

Although PANDAS and PANS cases were excluded, they were included in the search since PANDAS/PANS are often used as generic terms for secondary obsessive-compulsive syndromes. Duplicates of the retrieved references were removed using Citavi reference management software (Swiss Academic Software, Wädenswil, Switzerland) as well as the CADIMA Evidence Synthesis tool and database (Julius Kühn-Institut, Quedlinburg, Germany, https://www.cadima.info/). The initial results of 2384 publications were subjected to a detailed title and abstract screening by KR and NS. A full-text analysis was conducted for publications that met the eligibility criteria. Based on the extracted data, only cases with obsessive-compulsive syndromes or OCS in which brain involvement could be hypothesized were analyzed. This was the case if there were clear central nervous system (CNS) disorders associated with the OCS (e.g., Parkinson’s disease), if there was a peripheral disease associated with CNS symptoms (e.g., acanthocytosis or Fahr’s disease, common genetic basis), if the diagnostic findings indicated CNS involvement (e.g., pathological brain imaging findings or inflammatory CSF constellations), or after head trauma. In total, 189 publications consisting of 167 case reports and 22 case series, with 228 cases overall, were included (see Fig. 1).

### Data extraction and statistical analysis

The following data were extracted from the publications: sex, age, age at onset of OCS, clinical aspects of mainly mental disorders, severity of disease measured by Yale–Brown Obsessive–Compulsive Scale (Y-BOCS) [18], therapeutic response, laboratory findings of blood, cerebrospinal fluid (CSF), and genetic analyses, as well as electroencephalography (EEG), magnetic resonance imaging (MRI), computer tomography (CT), single photon emission computed tomography (SPECT), and positron emission tomography (PET) findings. If described, the affected brain region was noted. The cases were classified into brain lesions, genetic syndromes, head trauma, autoimmune-inflammatory processes, tumors, neurodegeneration, seizures, pathogens, and metabolic or other disorders. Given that tumors, for example, could also cause OCS through recurrent epileptic seizures, the classification named by the case report authors as the most prominent was used. The presentation of the findings was mainly descriptive. Figures were created using the R package ggplot2 [19, 20] and Adobe Illustrator (Adobe Inc., San José, CA).

## Results

### Search results

**Fig. 2:**
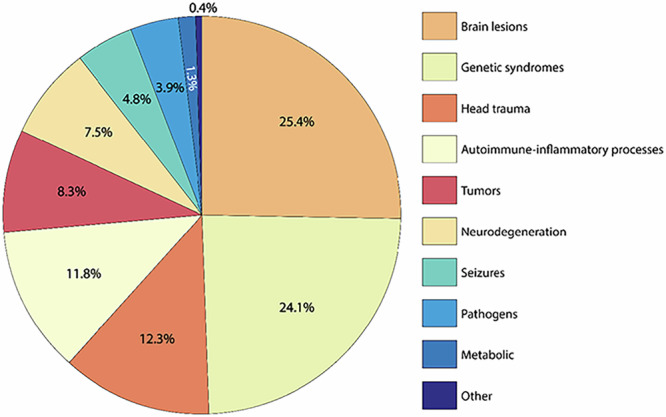
Proportion of cases with suspected secondary obsessive-compulsive syndromes by causes.

**Fig. 3:**
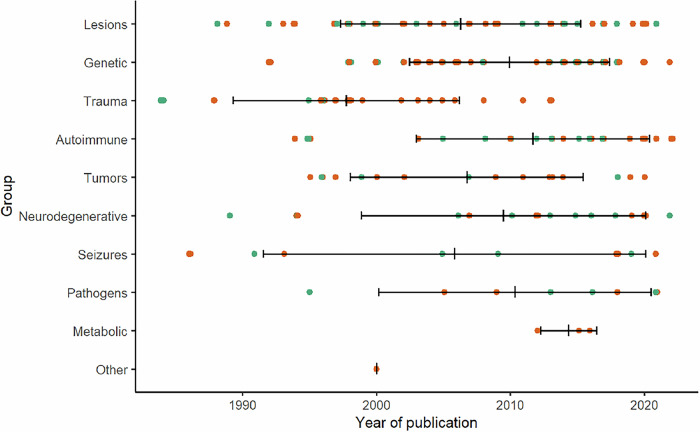
Year of publication of cases with suspected secondary obsessive-compulsive syndromes stratified by causes (n = 228). Female patients are plotted in turquoise and male patients in orange. Mean value is plotted as crossbar and error bars indicate ± one standard deviation.

**Fig. 4:**
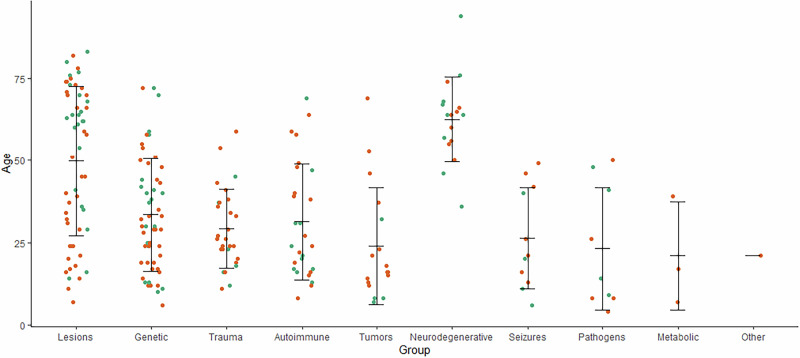
Reported age of cases with suspected secondary obsessive-compulsive syndromes by causes (n = 226). Female patients are plotted in turquoise and male patients in orange. Mean value is plotted as crossbar and error bars indicate ± one standard deviation.

**Fig. 5:**
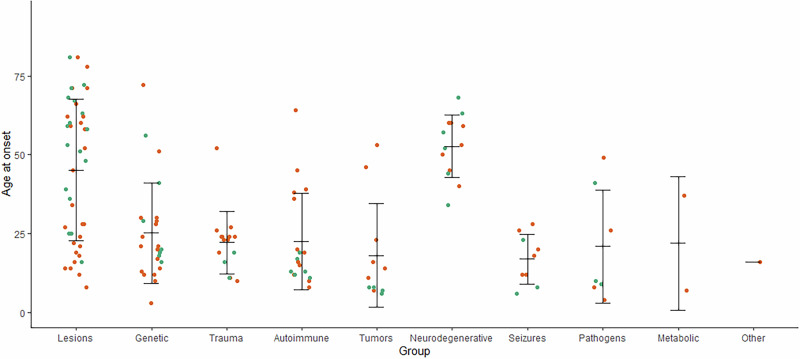
Reported age of onset of cases with suspected secondary obsessive-compulsive syndromes by causes (n = 143). Female patients are plotted in turquoise and male patients in orange. Mean value is plotted as crossbar and error bars indicate ± one standard deviation.

Overall, 228 cases of suspected secondary obsessive-compulsive syndromes were identified. Most cases reported were associated with brain lesions (25.4%), genetic disorders (24.1%), head trauma (12.3%), and inflammatory processes (11.8%). Figure 2 and Table 2 provide an overview of all detected causes. In terms of publication year, cases with head trauma tended to be from a longer time ago (mean publication year 1997.8 ± 8.4) with the last case published in 2013, while cases with autoimmune (mean publication year 2011.7 ± 8.7) or metabolic disorders (mean publication year 2014.3 ± 2.1) tended to be more recent (Fig. 3). Most patients were male (n = 146, 64.0%) and of all age categories (mean 37.3 ± 21.2 years, range 4–94 years, n = 226; Fig. 4). The onset of the disease/symptoms was mainly reported to have occurred around five years earlier (mean 31.7 ± 20.8 years, range 3–81 years, n = 143; Fig. 5). Regarding diagnostic testing, in 81.1% of the cases, diagnostic alterations in blood, EEG, and/or MRI/CT were found. At the same time, all cases with CSF and SPECT/PET alterations also had alterations in the diagnostics mentioned above. In 7.0% of the cases, diagnostic investigations were normal, and 11.8% did not report specific testing. Most often, abnormalities were found in brain imaging (MRI/CT; 66.2% of all cases, 84.8% of cases with reported imaging results, n = 179) and laboratory (23.9% of all cases, 55.1% of cases with reported laboratory testing, n = 98) diagnostics. In cases that reported the region of brain imaging alterations in detail (n = 102; 43.2%), the frontal lobe (34.3%), the basal ganglia (26.5%), and the temporal lobe (21.6%) were mostly affected. Volume loss (26.8%) and cerebral infarction (19.6%) were predominant in these cases. Regarding laboratory analyses, genetic (30.6%) and immunological (14.3%) alterations were mostly described. EEG changes were identified in 42.9% of cases with reported EEG results (n = 49, EEG alterations in 9.3% of all cases), CSF alterations in 40.7% of cases with reported CSF results (n = 27, CSF alterations in 4.9% of all cases), and PET/SPECT findings in 72.7% of cases with reported PET/SPECT results (n = 11, PET/SPECT alterations in 3.6% of all cases). Of all the cases that reported therapy (79.4%, n = 181), an improvement was reported in 71.3%, while the remaining 28.7% did not clearly benefit. For therapy success, selective serotonin reuptake inhibitor (SSRI) treatment (16.6%) and a causal treatment depending on the underlying disease (11.6%) were common. When combinations of treatments were considered, causal treatment was a part of 27.1% of all successful treatments. A detailed overview can be found in Supplementary Table 1.

**Table 2:** Summary of 228 cases with suspected secondary obsessive-compulsive syndromes by causes.

**Brain lesions (n** = **58)**	Infarction (48.3%) Lesions due to neurosurgeries (15.5%) Cerebral compression due to cysts and hydrocephalus (12.1%) Haemorrhage (5.2%) Late-life reactivation of OCS associated with lesions (5.2%) Lesions of unclear origin (3.4%) Focal striatal abnormalities (3.4%) Vascular encephalopathy (1.7%) White matter hyperintensities (1.7%) Schizencephaly (1.7%) Cavernous sinus thrombosis (1.7%)
**Genetic syndromes (n** = **55)**	Huntington’s disease (14.5%) Idiopathic basal ganglia calcification (9.1%) Tuberous sclerosis (7.3%) Pantothenate kinase-associated neurodegeneration and neurodegeneration with brain iron accumulation (7.3%) Neuroacanthocytosis (7.3%) Down syndrome (7.3%) Niemann-Pick disease (type C) (5.5%) Wilson’s disease (5.5%) Prader-Willi-/Angelman-Sachs syndrome (3.6%) MELAS (mitochondrial disease) (3.6%) Velocardiofacial (22q11 deletion) syndrome (3.6%) Kleefstra syndrome (1.8%) Myoclonus-dystonia syndrome (1.8%) SHANK2 variant (1.8%) Facioscapulohumeral muscular dystrophy (1.8%) Johanson-Blizzard syndrome (1.8%) 9q33.1 deletion (1.8%) Wolfram syndrome (1.8%) Joubert syndrome (1.8%) Adult-onset X-linked adrenoleukodystrophy (1.8%) Spinocerebellar ataxia 12 (1.8%) De novo balanced translocation (2;10)(q24;q22) (1.8%) 48 XXYY syndrome (1.8%) Acute porphyria (1.8%) PCDH19 gene variant mosaicism (1.8%)
**Head trauma (n** = **28)**	Traffic accident (64.3%) Assault (14.3%) Industrial accidents (10.7%) Rock-climbing accident (3.6%) Not specified (7.1%)
**Autoimmune-inflammatory (n** = **27)**	Multiple sclerosis and other chronic inflammatory CNS diseases (33.3%) Autoimmune encephalitis-like presentations (33.3%) Systemic lupus erythematosus (14.9%) Sjögren’s syndrome (7.4%) Primary antiphospholipid syndrome (3.7%) Melkersson-Rosenthal syndrome (3.7%) Miller Fischer syndrome (3.7%)
**Tumors (n** = **19)**	Glioma (42.1%) Germinoma (36.8%) Cerebellar tumor (5.3%) Meningioma (5.3%) Acoustic neuroma (5.3%) Pontine mass (5.3%)
**Neurodegenerative disorders (n** = **17)**	Frontotemporal dementia (58.8%) Alzheimer’s disease (11.8%) Dementia with Lewy Bodies (11.8%) Parkinsons’s disease (5.9%) Progressive supranuclear palsy (5.9%) Amyotrophic lateral sclerosis (5.9%)
**Seizures (n** = **11)**	Temporal lobe epilepsy (36.4%) Generalised tonic–clonic epilepsy (36.4%) Focal impaired awareness/absence seizures (18.2%) Panayiotopoulos syndrome (9.1%)
**Pathogen-related (n** = **9)**	Human immunodeficiency virus (33.3%) Mycoplasma pneumonia (22.2%) Cerebral malaria (11.1%) Herpes (11.1%) Varicella (11.1%) Powassan virus (11.1%)
**Metabolic (n** = **3)**	Coenzyme Q10 deficiency (33.3%) Vitamin B12 deficiency (33.3%) Vitamin D deficiency (33.3%)
**Other (n** = **1)**	Langerhans cell histiocytosis (100.0%)

*MELAS*, mitochondrial encephalomyopathy, lactic acidosis and stroke-like episodes.

### Suspected secondary obsessive-compulsive syndrome cases categorized by causes

#### Brain lesions

Brain lesions not caused directly by tumors or head trauma (see below) accounted for 25.4% of all patients (n = 58). The majority of reported patients (48.3%, n = 28) were due to cerebral infarctions. Among individuals with infarction, onset of OCS predominantly occurred in elderly people (mean age 63.7 ± 16.1, range 21–83 years; reported onset 59.7 ± 16.5, range 18–81 years, n = 24), whereas in all other brain lesion categories (n = 30, Table 2), OCS also developed among younger patients (mean age 36.4 ± 20.3, range 7–73 years; mean onset 26.8 ± 13.73, range 8–59 years, n = 19). In 39.6% of all brain lesion cases with an indicated affected brain region, the basal ganglia were specified, and in almost a third (31.3%), the frontal lobe was indicated. Therapeutic interventions comprised conventional OCD treatments (SSRI, clomipramine, and cognitive behavioral therapy [CBT]). In 84.5% of the cases, treatments were described, of which 71.4% reported successful therapy. Of particular interest is the case series by Salinas et al. [21], which represents three patients whose OCD remitted in adolescence but recurred due to infarction, hemorrhage, and cerebral hypoxia in later life.

#### Genetic syndromes

Genetic syndromes accounted for 24.1% of the cases described (n = 55). The group of genetic diseases was very heterogeneous and included a wide variety of diseases with different pathomechanisms and age groups (mean 33.4 ± 17.2 years, range 6–72 years; age of “onset” 25.3 ± 15.9, range 3–72 years, n = 24). Notably, 47.0% of the cases described were associated with genetic syndromes affecting basal ganglia function. In cases with reported imaging diagnostics (MRI/CT; 56.4%, n = 31), 87.1% indicated alterations, and only 12.9% had a normal brain scan. Among cases that reported treatment (65.5%, n = 36), treatment success was described in 77.8% (n = 28) with mostly SSRI therapy. In general, therapies involving conventional OCD therapy (SSRI, clomipramine, and CBT) improved symptoms by 57.6% and even led to a slight improvement by another 15.2%. Causal treatments for many diseases are rare, but disease-specific treatment was reported to be helpful in 11.2% of cases.

***Huntington’s disease*** was documented in eight patients with variations noted in symptom onset across patients. Half of the patients reported OCS before disease onset, while the other half developed OCS concurrently with the onset of Huntington’s disease, and even a decade later in one case [22].

***Idiopathic basal ganglia calcification***, also known as Fahr’s disease, was found in five patients across all age groups. Basal ganglia calcification was confirmed by CT, but in only one case an underlying gene mutation (SLC20A2) [23] was described. No calcium, phosphate or parathyroid hormone abnormalities were reported in any of the cases, although no information was available in two cases.

***Neurodegeneration with brain iron accumulation***, as seen in pantothenate kinase-associated neurodegeneration, was described in four patients who all developed OCS before the age of 20 and showed MRI alterations, such as the “eye of the tiger” sign typical of iron accumulation in the globus pallidus.

***Tuberous sclerosis*** was observed in four patients from infancy to the late 20 s, with acute onset or slow development over several months. Seizure control with an anticonvulsant played an important role in the treatment of most cases. However, OCS continued to improve with SSRI or clomipramine therapy in most patients.

***Neuroacanthocytosis*** caused OCS in four patients from childhood until their early 20 s, all exhibiting basal ganglia alterations observable on MRI.

***Down Syndromes with OCS*** was reported for three male and one female patient [24]. Two presented with comorbid depression and two with aggression-related problems.

***Niemann-Pick disease (type C)*** was identified in three patients in their mid-twenties [25, 26].

***Wilson’s disease*** was reported in two patients [27, 28] and another patient with a related mutation [29]. One case of a 17-year-old male developed OCS within one month and did not respond to SSRI treatment [27] but to causal Morbus Wilson treatment with D-penicillamine and a low-copper diet. In contrast, a 12-year-old male developed OCS during causal Morbus Wilson treatment and responded to SSRI treatment [28].

***Prader-Willi/Angelmann-Sachs*** syndromes associated with OCS were observed in two young girls.

***Velocardiofacial (22q11 deletion) syndrome*** was described in two males [30].

***Other genetic syndromes***, such as MELAS [31] and acute porphyria [32] were reported in 18 other young to middle age patients.

#### Head trauma

Head trauma was associated with 12.3% of all included cases. It was mainly induced by traffic accidents (64.3%). The group consisted of different ages (11–59 years; mean age 29.2 ± 11.9), who mainly experienced closed head trauma without the specification of affected brain regions (50.0%). Interestingly, most patients were investigated by brain imaging (MRI/CT, 85.7%), with 37.5% showing normal findings. However, when an affected brain region was indicated, it was mainly in the frontal lobe (85.7%) and/or temporal lobe (50.0%). The therapy response was mixed, with 76.2% of those with reported therapy benefiting from treatment (n = 21, 75.0%).

#### Autoimmune-inflammatory processes

In total, 27 cases (11.8%) with autoimmune-inflammatory etiologies of OCS were identified across patients of varying ages (mean age 31.3 ± 17.5, range 8–69 years). Brain involvement was determined to be possible in 25.9%, and probable in 74.1% of the cases. The most prevalent conditions included multiple sclerosis and other chronic inflammatory CNS diseases (33.3%) and autoimmune encephalitis-like presentations (33.3%), followed by rheumatologic diseases such as systemic lupus erythematosus (14.9%), and Sjögren’s syndrome (7.4%). Immunotherapy was administered in 22 cases, with 11 (50.0%) patients benefiting from it. In general, treatment response (n = 18, 69.2%) was reported for a causal treatment of the autoimmune-inflammatory disease in 37.0% and an OCD-specific pharmacotherapy in 18.5%.

***Multiple sclerosis and other chronic inflammatory CNS diseases*** in the context of OCS were described in nine cases (six males and three females). Several patients received steroid treatment with regression of the neurological symptoms but not of the OCS [33, 34]. Only in one case was a questionable effect on OCD observed, but clomipramine treatment was started at the same time [35]. However, SSRI therapy seemed to improve OCS in four patients [33, 34, 36, 37].

***Autoimmune encephalitis-like presentations*** were identified with CNS antibodies against LGI1 [38], GAD65 [39], CV2 [40] and Ma2 [41]. In a case of suspected Hashimoto encephalopathy [42], thyroid antibodies were observed. Also, one case of seronegative encephalitis [43] and another with paraneoplastic limbic encephalitis associated with a mediastinal seminoma [44] were described. In two cases, antibody testing on murine brain slices identified novel anti-basal-ganglia antibodies in serum and CSF [10] and anti-cytoplasmatic antibodies in serum [45] associated with OCS, with the underlying antigen unknown. Inflammatory signs in serum, CSF, MRI, EEG, or FDG-PET were found in seven of the nine cases (67.0%). Under immunotherapy, OCS fully regressed in three cases, improved to different degrees in three other cases, did not improved in two cases, and was not tried in one case.

***Systemic lupus erythematosus*** has been reported in three females and one male with OCS during adolescence and early adulthood [46–49]. The patients presented with a mixed neuropsychiatric phenotype with additional psychotic symptoms. Immunotherapy with corticosteroids resulted in improvement in one case [48] and was suspected to be the cause of the symptoms in two cases [47, 49].

***Sjögren’s syndrome*** was documented in two adolescent females who both benefited from immunotherapy [50, 51].

***Other autoimmune disorders***, such as anti-phospholipid syndrome [52], Melkersson-Rosenthal Syndrome [53], and Miller‑Fisher syndrome [54], were reported as being associated with OCS.

#### Tumors

***Tumors*** were associated with secondary obsessive-compulsive syndromes in 8.3% (n = 19) affecting patients of young age (mean 24.0 ± 17.7 years, range 7–69 years, n = 17; mean onset 18.1 ± 16.4 years, range 6–53 years, n = 11). Among these cases, gliomas were the most prevalent (42.1%), followed by germinomas (36.8%). The affected brain regions were very distinct, with two patients each identified for the temporal lobe, basal ganglia, pineal gland, and midbrain. Treatment was reported in 68.4% of the cases, with 53.8% showing success.

**Gliomas** affected patients across a broader age spectrum ranging from 8–69 years. Treatment primarily focused on tumor therapy and overall patient survival, yielding mixed treatment responses.

**Germinomas** predominantly affected young patients between 7 and 23 years of age. OCS often emerged months to years after tumor diagnosis or treatment. One patient developed OCS directly with the growth of a pineal germinoma, which did not regress with tumor removal [55]. Another patient benefited from psychopharmacological treatment, but with tumor relapse, the OCS exacerbated [56].

#### Neurodegenerative disorders

Neurodegenerative disorders were associated with 7.5% of the identified cases (n = 16). Brain involvement was probable in almost all cases (93.8%). Elderly people were predominantly affected (mean age 62.4 ± 13.3 years, range 36–94 years), with the onset mostly reported ten years earlier (mean age 52.9 ± 10.2 years, range 34–68 years, n = 12). The most frequent neurodegenerative disorder described was frontotemporal dementia (FTD; 56.3%). The benefits of psychotherapeutic and pharmaceutical treatment were reported in 41.7% of the patients.

***FTD*** was described in ten cases with mostly late-onset OCS, but the youngest patient was in her mid-thirties [57]. In all cases, frontal, temporal, or frontotemporal brain involvement was confirmed on MRI or CT. In one case, the OCS responded to therapy with sertraline and cyamemazine [58]. In another, a slight symptom reduction under trazodone was reported [59], whereas other patients showed no improvement with medication and/or psychotherapy. The case reported by Donde et al. [58] was of particular interest because reactivation of OCS after a period of 15 years without symptoms was associated with the onset of FTD.

***Alzheimer’s disease*** in the context of OCS was described in two female patients [60, 61], with no observed therapeutic benefit from medication.

***Dementia with Lewy bodies*** was described in two male OCD patients in their sixties [62]. Both showed improved OCS with rivastigmine, although one also received sertraline and levodopa.

***Parkinson’s disease*** was reported in one OCD case of a man in his seventies [63]. No improvement under levodopa/carbidopa was observed.

***Other disorders*** associated with OCS included progressive supranuclear palsy [64] and amyotrophic lateral sclerosis [65].

#### Seizures

A total of eleven patients (4.8%) presented with epileptic seizures in association with OCS. Predominantly, these patients were within the first half of life (age range 6–49 years; mean age 26.4 ± 15.3 years). Temporal lobe epilepsy (36.4%) and generalized tonic-clonic seizures (36.4%) were most frequently reported. Therapeutically, the main focus was good seizure control by neurosurgical measures and anticonvulsant medication. In these cases, in addition to good seizure control, a decrease in OCS was observed in eight patients (72.7%). In one case, CBT improved OCS [66], but in two other cases, no relevant improvement was observed [66, 67]. In one case [68], treatment with SSRIs and clomipramine was described but did not lead to improvement until anticonvulsive treatment was started. In only one case (9.1%) no improvement was observed [67].

#### Pathogen-related

In our search algorithm, 63 case reports and case series with PANS/PANDAS were identified and excluded from the analysis (see Methods). Nevertheless, nine other cases showed pathogen-related OCD that did not clearly align with the PANDAS/PANS diagnostic criteria (e.g., due to known neurological or medical disorder associated with the symptoms, such as severe viral encephalitis) and are discussed here (mean age 23.1 ± 18.6 years, range 4–50 years). Most cases were subjected to MRI or CT (77.8%), of which 85.7% showed alterations. This includes, for example, HSV encephalitis [69] or infections with malaria [70], Varicella zoster [71] or HIV infection [72, 73].

#### Metabolic and other diseases

Most metabolic causes of OCS were reported in genetic disorders of metabolism due to the deposition of iron, copper, defective proteins, or others (see Section 3.2.2). Furthermore, three cases (1.4%) of OCS related to vitamin deficiency (vitamin D, coenzyme Q10 and vitamin B12) were described. In one case of vitamin B12 deficiency in a man in his late 30 s, a significant improvement of OCS was reported with intramuscular methylcobalamin supplementation [74]. A case of coenzyme Q10 deficiency was found to have OCS, in addition to myopathy and growth retardation [75]. A 7-year-old boy with vitamin D deficiency and streptococcal antibodies was remitted with vitamin D and iron supplementation [76].

Furthermore, one case of a 21-year-old man with Langerhans cell histiocytosis and reduced cerebellar perfusion did not fit any previous classification [77].

## Discussion

In this systemic review, 228 cases with suspected secondary obsessive-compulsive syndromes and various associated causes were identified.

The median age of the published cases was 37.3 years, ranging from 4 to 94 years. The mean age of first onset (when described) was 31.7 years. Overall, this is later, on average, than for primary OCD. The onset of primary OCD was described as 19.5 years [2]. However, the mean age detected also shows that secondary obsessive-compulsive syndromes are by no means exclusively late-onset manifestations, because the majority of cases occurred at a similar age as primary OCD. A total of 64% of the patients with suspected secondary causes were male, which is in contrast to the slightly higher numbers of females with primary OCD [2, 3].

The most common causes of secondary obsessive-compulsive syndromes were brain lesions, genetic syndromes, head trauma and autoimmune inflammatory processes. These four causes accounted for 73.6% of all published cases. However, there is a notable difference between the publication years of these groups. While head trauma used to be reported more frequently in the past (mean publication year 1998), autoimmune causes, as rather novel discoveries, have been increasingly published in recent years (mean publication year 2012) and it could be expected that their significance will become increasingly important due to possible therapeutic consequences. Furthermore, the causal relationships varied across the four entities. For example, there is no uniform spectrum of associated CNS antibodies (similar to patients with psychosis; [78]). Overall, however, the spectrum was much broader, including tumors, neurodegeneration, seizures, pathogens, metabolic and other disorders. This emphasizes the heterogeneous etiology of secondary obsessive-compulsive syndromes indicating that different mechanisms might lead to the occurrence of OCS. In particular, (mono)genetic syndromes, in which the molecular causes can be understood in detail, could provide exciting insights into the fundamental pathophysiological mechanisms (cf. [79]) and raise hopes for research into the molecular basis of OCS. Most of the identified cases showed alterations in diagnostic testing using brain imaging with MRI or CT (in 66.2%). This supports the importance of brain imaging for patients with OCS. However, other diagnostic tools (e.g., an EEG) were not regularly performed, and laboratory analyses of the blood were variable. In brain imaging, pathologies were most commonly reported in the frontal lobe (34.3%) and basal ganglia (26.5%). This supports the common neurostructural hypotheses of OCD that postulate cortico-striato-thalamo-cortical circuit dysfunction [3]. Of note, different circuits are thought to be linked to the development and maintenance of OCD symptoms [80]. Whether the lesions reported in the cases of this review can be considered strategic lesions, i.e. lesions with a high and specific risk for the development of OCD, remains to be answered. Moreover, it may be less the lesion itself and more its functional embedding within disease-relevant networks that may determine its clinical significance. Accordingly, an analysis of the connectivity profile of these lesions may provide more insight into whether they are part of OCD-related circuits. A similar network-based approach was recently applied to tic disorders by Ganos et al. [81] and Baldermann et al. [82]. Interestingly, some of the lesions that we identified in OCD cases in this review partially overlap with the tic disorder lesion network, suggesting a possible neuroanatomical convergence of secondary forms of both disorders, while other lesion locations may be more specific to OCD. Future studies may thus want to aim at performing a specific lesion network analysis for OCD to better understand the link between lesion location and symptoms.

Several cases received causal therapies. Around one-third of patients benefited from causal therapies, including combination therapies. This shows the therapeutic importance of searching for secondary causes. For example, for autoimmune causes, there are direct therapeutic consequences through alternative treatment options with immunotherapies (cf. [10, 12]). However, anticonvulsants for epilepsy [67, 68, 83–86], surgical resection of underlying tumors [87], treating underlying hydrocephalus [88–90], low copper diet and D-Penicillamin in Wilson’s Disease [27] or substituting underlying vitamin deficiencies [74, 76] were also reported as successful causal treatments. Regardless of the causal treatment, secondary causes can play an important role in an improved understanding of the disease in developing an individual bio-psycho-social model. Therefore, they might contribute to destigmatization of the affected patients and their caregivers due to valid self-concepts.

These developments are addressed in the international diagnostic classification systems DSM-5 and ICD-11, and a disorder category for secondary obsessive-compulsive syndromes has been established. However, leading international OCD guidelines, such as the American Psychiatric Association guideline (from 2007, updated in 2013), the British NICE guideline (from 2005), and the German DGPPN S3 OCD guideline (from 2022), do partly not yet contain specific recommendations for organic clarification due to a lack of data (Table 1; [7–9]).The American guideline recommends an assessment of general medical history, and the German DGPPN S3 guideline advises for a somatic and neurological examination at initial presentation to rule out somatic diseases [7, 9]. Specific recommendations for somatic assessment can only be found in the DGPPN S3 guidelines for late-onset OCD (onset of symptoms after the age of 50), which advocate for neuropsychological screening and brain imaging (CT or better MRI) [9]. The findings of this review favor broader diagnostic approaches in late onset OCD, but also in younger patients as the average age of most cases is well below 50. With the Freiburg diagnostic protocol (FDP) for patients with OCD (“FDP-OCD”), which also includes further diagnostic investigations, such as a lumbar puncture or a genetic assessment, in the case of specific abnormalities, indications of an organic cause were detected in 16% of the multimodal diagnostic work-up in a pre-selected inpatient cohort of 61 severely ill patients [91]. Larger studies utilizing multimodal diagnostic approaches, such as FDP-OCD, in large, unselected patients with OCD are necessary to determine the number of secondary obsessive compulsive syndromes and the optimal diagnostic approach. Based on these results, recommendations for guidelines can then be formulated. The preliminary data from this review currently suggest an important role for MRI in a variety of cases with secondary OCD.

This systematic literature review has obvious limitations due to the heterogeneous case reports with varying quality and depth of detail. The reported cases were subjectively described from the point of view of the respective practitioners, and no overarching standardized criteria were applied for diagnostic criteria, organic diagnostics, or treatment. In particular, the data on treatment response should be interpreted with caution. Reported therapy effects in case studies may be influenced by placebo effects, given the inherent limitations in applying a randomized and blinded methodology. Furthermore, there may have been a bias regarding which cases were reported and published (e.g., more patients with a positive outcome might have been published). This includes a possible gender bias, as clinical case reports tend to be more frequently published for male patients [92]. A further bias results from the fact that some causes were reported more frequently a longer time ago, e.g. head trauma, while other causes such as autoimmune processes have gained importance as a newer field of research in recent years. Overall, each case study has a high risk of bias, yet a risk of bias assessment of each case report was not carried out here. PANDAS/PANS patients were omitted, as an association with OCS has been described for some time and has already been established [15]. This underestimates the role of pathogen-induced autoimmunity and the relevance of inflammation-related diagnostics in this article. Another critical issue is the distinction between correlation and causality. It is sometimes difficult to prove that OCS was caused by an observed condition, such as a tumor or brain lesion, and, conversely, that it was not causal and merely coincidental. ICD-11 emphasizes that the clinical presentations of primary and secondary clinical presentations may be similar. Still, it should be assessed whether the potentially explanatory medical condition is known to cause OCS and whether there is a temporal relationship between the condition and the symptoms [5]. Furthermore, atypical clinical features, such as late age of onset, sudden appearance of symptoms, accompanying cognitive impairment or focal neurological signs, can point towards secondary obsessive-compulsive syndromes [5]. Nevertheless, clear-cut diagnostic criteria are lacking. Although we attempted to include only patients with possible brain involvement, some false-positive cases may clearly have been included in our search (especially if no associated brain pathologies were identified in the diagnostic work-up). Broad inclusion criteria were deliberately chosen here to demonstrate the possible spectrum of organic causes for OCS. However, a broader and more all-encompassing search strategy might have detected even more cases.

## Conclusions

A variety of potential causes for the development of secondary obsessive-compulsive syndromes have been reported in the literature. Accordingly, practitioners should consider brain lesions, genetic syndromes, trauma, autoimmune-inflammatory processes, tumors, neurodegeneration, seizures, pathogens, and metabolic or other disorders for secondary OCS. In addition to a thorough physical examination and medical history, laboratory analyses and MRI supported the identification of secondary causes and, in some cases, paved the way for alternative therapies. Larger studies are needed to determine the prevalence of secondary obsessive-compulsive syndromes, the best possible diagnostic approach for their detection, and the most promising causal treatment approaches.

## Supplementary information

Supplementary Table 1

## Data Availability

All relevant findings are presented descriptively in the paper and supplementary material.
