# Supplementary material for: Secondary obsessive-compulsive syndromes: a systematic literature review resulting in 228 suspected cases
Source: Mol Psychiatry. 2025 Dec 23;31(2):1121–31. doi: 10.1038/s41380-025-03395-1 (PMC12815680; doi:10.1038/s41380-025-03395-1)
Supplement: Supplementary file 1 — Supplementary Table 1 [file 41380_2025_3395_MOESM1_ESM.pdf]

	All (n=228)	Brain lesions (n=58)	Genetic syndromes (n=55)	Head trauma (n=28)	Autoimmune-inflammatory processes (n=27)	Tumors (n=19)	Neurodegenerative disorders (n=17)	Seizures (n=11)	Pathogen-related (n=9)	Metabolic (n=3)	Other (n=1)
**Subgroup types**	Brain lesions: 25.4%<br>Genetic syndromes: 24.1%<br>Head trauma: 12.3%<br>Autoimmune-inflammatory processes: 11.8%<br>Tumors: 8.3%<br>Neurodegenerative disorders: 7.5%<br>Seizures: 4.8%<br>Pathogen-related: 3.9%<br>Metabolic: 1.3%<br>Other: 0.4%	Infarction (48.3%)<br>Lesions due to neurosurgeries (15.5%)<br>Cerebral compression due to cysts and hydrocephalus (12.1%)<br>Hemorrhage (5.2%)<br>Late-life reactivation of OCS associated with lesions (5.2%)<br>Lesions of unclear origin (3.4%)<br>Focal striatal abnormalities (3.4%)<br>Vascular encephalopathy (1.7%)<br>White matter hyperintensites (1.7%)<br>Schizencephaly (1.7%)<br>Cavernous sinus thrombosis (1.7%)	Huntington's disease (14.5%)<br>Idiopathic basal ganglia calcification (9.1%)<br>Tuberous sclerosis (7.3%)<br>Pantothenate Kinase-associated neurodegeneration and neurodegeneration with brain iron accumulation (7.3%)<br>Neuroacanthocytosis (7.3%)<br>Down syndrome (7.3%)<br>Niemann-Pick disease (type C) (5.5%)<br>Wilson's disease (5.5%)<br>Prader-Willi-/Angelman-Sachs syndrome (3.6%)<br>MELAS (mitochondrial disease) (3.6%)<br>Velocardiofacial (22q11 deletion) syndrome (3.6%)<br>Kleefstra syndrome (1.8%)<br>Myoclonus-dystonia syndrome (1.8%)<br>SHANK2 variant (1.8%)<br>Facioscapulohumeral muscular dystrophy (1.8%)<br>Johanson-Blizzard syndrome (1.8%)<br>9q33.1 deletion (1.8%)<br>Wolfram syndrome (1.8%)<br>Joubert syndrome (1.8%)<br>Adult-onset X-linked adrenoleukodystrophy (1.8%)<br>Spinocerebellar ataxia 12 (1.8%)<br>De novo balanced translocation (2;10)(q24;q22) (1.8%)<br>48 XXYY syndrome (1.8%)<br>Acute porphyria (1.8%)<br>PCDH19 gene variant mosaicism (1.8%)	Traffic accident (64.3%)<br>Assault (14.3%)<br>Industrial accidents (10.7%)<br>Rock-climbing accident (3.6%)<br>Not specified (7.1%)	Multiple sclerosis and other chronic inflammatory CNS diseases (33.3%)<br>Autoimmune encephalitis-like presentations (33.3%)<br>Systemic lupus erythematosus (14.9%)<br>Sjogren's syndrome (7.4%)<br>Primary antiphospholipid syndrome (3.7%)<br>Melkersson-Rosenthal-syndrome (3.7%)<br>Miller Fisher syndrome (3.7%)	Glioma (42.1%)<br>Germinoma (36.8%)<br>Cerebellar tumor (5.3%)<br>Meningioma (5.3%)<br>Acoustic neuroma (5.3%)<br>Pontine mass (5.3%)	Frontotemporal dementia (58.8%)<br>Alzheimer's disease (11.8%)<br>Dementia with Lewy Bodies (11.8%)<br>Parkinsons's disease (5.9%)<br>Progressive supranuclear palsy (5.9%)<br>Amyotrophic lateral sclerosis (5.9%)	Temporal lobe epilepsy (36.4%)<br>Generalized tonic–clonic epilepsy (36.4%)<br>Focal impaired awareness/absence seizures (18.2%)<br>Panayiotopoulos syndrome (9.1%)	Human immunodeficiency virus (33.3%)<br>Mycoplasma pneumoniae (22.2%)<br>Cerebral malaria (11.1%)<br>Herpes (11.1%)<br>Varicella (11.1%)<br>Powassan virus (11.1%)	Coenzyme Q10 deficiency (33.3%)<br>Vitamin B12 deficiency (33.3%)<br>Vitamin D deficiency (33.3%)	Langerhans cell histiocytosis (100.0%)
**Year of publication (mean ± SD (range)) in years**	2007.2±9.7 (1984–2022) (n=228)	2006.3±9.0 (1988–2021) (n=58)	2009.9±7.5 (1992–2022) (n=55)	1997.8±8.4 (1984–2013) (n=28)	2011.7±8.7 (1994–2022) (n=27)	2006.7±8.7 (1995–2020) (n=19)	2009.5±10.6 (1989–2022) (n=17)	2005.8±14.2 (1986–2021) (n=11)	2010.3±10.2 (1995–2021) (n=9)	2014.3±2.1 (2012–2016) (n=3)	2000 (n=1)
**Age (mean ± SD (range)) in years**	37.3±21.2 (4-94) (n=226)	49.7±22.8 (7-83) (n=58)	33.4±17.2 (6-72) (n=55)	29.2±11.9 (11-59) (n=28)	31.3±17.5 (8-69) (n=27)	24.0±17.7 (7-69) (n=17)	62.5±12.9 (36-94) (n=17)	26.4±15.3 (6-49) (n=11)	23.1±18.6 (4-50) (n=9=	21.0±16.4 (7-39) (n=3)	21 (n=1)
**Age at onset in years**	31.7±20.8 (3-81) (n=143)	45.2±22.4 (8-81) (n=43)	25.3±15.9 (3-72) (n=24)	22.2±9.9 (10-52) (n=15)	22.6±15.3 (8-64) (n=18)	18.1±16.4 (6-53) (n=11)	52.7±9.8 (34-68) (n=13)	17.0±7.9 (6-28) (n=9)	21.0±17.9 (4-49) (n=7)	22.0±21.2 (7-37) (n=2)	16 (n=1)

**Age at onset category**	<20 years: 82 (36.0%)<br>20-34 years: 41 (18.0%)<br>35-49 years: 24 (10.5%)<br>50-64 years: 31 (13.6%)<br>>=65 years: 14 (6.1%)<br>(n=192)	<20 years: 12 (22.6%)<br>20-34 years: 10 (18.9%)<br>35-49 years: 5 (9.4%)<br>50-64 years: 14 (30.2%)<br>>=65 years: 10 (18.9%)<br>(n=53)	<20 years: 21 (51.2%)<br>20-34 years: 12 (29.3%)<br>35-49 years: 3 (7.3%)<br>50-64 years: 3 (7.3%)<br>>=65 years: 2 (4.9%)<br>(n=41)	<20 years: 8 (34.8%)<br>20-34 years: 9 (39.1%)<br>35-49 years: 4 (17.4%)<br>50-64 years: 2 (8.7%)<br>(n=23)	<20 years: 15 (68.2%)<br>20-34 years: 2 (9.1%)<br>35-49 years: 4 (18.2%)<br>50-64 years: 1 (4.5%)<br>(n=22)	<20 years: 12 (80.0%)<br>20-34 years: 1 (6.7%)<br>35-49 years: 1 (6.7%)<br>50-64 years: 1 (6.7%)<br>(n=15)	<20 years: 0 (0.0%)<br>20-34 years: 1 (6.7%)<br>35-49 years: 4 (26.7%)<br>50-64 years: 8 (53.3%)<br>>=65 years: 2 (13.3%)<br>(n=15)	<20 years: 6 (54.5%)<br>20-34 years: 5 (45.5%)	<20 years: 5 (62.5%)<br>20-34 years: 1 (12.5%)<br>35-49 years: 2 (25.0%)<br>(n=8)	<20 years: 2 (66.7%)<br>20-34 years: 1 (33.3%)	<20 years: 1 (100%)<br>(n=1)
**Sex**	81 female (35.5%)<br>146 male (64.0%)<br>(n=227)	23 female (39.7%)<br>35 male (60.3%)<br>(n=58)	20 female (36.4%)<br>35 male (63.6%)<br>(n=55)	6 female (21.4%)<br>22 male (78.6%)	11 female (40.7%)<br>16 male (59.3%)<br>(n=27)	4 female (22.2%)<br>14 male (77.8%)<br>(n=18)	9 female (52.9%)<br>8 male (47.1%)<br>(n=17)	4 female (36.4%)<br>7 male (63.6%)<br>(n=11)	4 female (44.4%)<br>5 male (55.6%)	0 female (0.0%)<br>3 male (100.0%)	0 female (0.0%)<br>1 male (100%)
**Y-BOCS**	24.9±7.8 (n=69)	23.8±8.4 (n=30)	27.1 ± 4.5 (n=11)	27.7±4.1 (n=7)	23.5±9.3 (n=10)	21.5±10.6 (n=2)	32.0±4.0 (n=3)	23.7±12.7 (n=3)	13.0±0 (n=1)	27.0±4.2 (n=2)	
**Patients with blood alterations**	Blood results reported n=98 (43.0%)<br>• 44 normal (44.9%)<br>• 30 genetic alterations (30.6%)<br>• 14 rheumatological/ immunological pathologies (14.3%)<br>• 12 patients with multiple alterations (12.2%)<br>• 7 routine hematological changes (7.1%)<br>• 5 infectious findings (5.1%)<br>• 5 trace element alterations (5.1%)<br>• 4 other findings (4.1%)<br>• 1 endocrinological change (1.0%)	Blood results reported n=18 (31.0%)<br>• 14 normal (77.8%)<br>• 3 routine hematological findings (16.7%)<br>• 2 infectious findings 11.1%)<br>• 1 genetic finding (5.6%)<br>• 1 other finding (5.6%)<br>• 1 patient with multiple alterations (5.6%)	Blood results reported n=40 (72.7%)<br>• 26 genetic findings (65.0%)<br>• 12 normal (30.0%)<br>• 4 trace element alterations (10.0%)<br>• 3 patients with multiple alterations (7.5%)<br>• 1 other finding (2.5%)	Blood results reported n=0	Blood results reported n=18 (66.7%)<br>• 12 rheumatological/ immunological findings (66.7%)<br>• 6 normal (33.3%)<br>• 1 routine hematological finding (5.6%)<br>• 1 genetic finding (5.6%)<br>• 1 infectious finding (5.6%)<br>• 1 patient with multiple alterations (5.6%)	Blood results reported n=2 (10.5%)<br>• 1 normal (50.0%)<br>• 1 endocrinological finding (50.0%)	Blood results reported n=8 (47.1%)<br>• 6 normal (75.0%)<br>• 2 genetic findings (25.0%)	Blood results reported n=1 (10.0%)<br>• 1 normal (100.0%)	Blood results reported n=6 (66.7%)<br>• 2 normal (33.3%)<br>• 2 routine hematological findings (33.3%)<br>• 2 rheumatological/ immunological findings (33.3%)<br>• 2 infectious findings (33.3%)<br>• 2 patients with multiple alterations (33.3%)	Blood results reported n=2 (66.7%)<br>• 1 routine hematological finding (50.0%)<br>• 1 trace element alteration (50.0%)<br>• 1 other finding (50.0%)<br>• 1 patients with multiple alterations (50.0%)	Blood results reported n=1 (100%)<br>• 1 normal (100.0%)
**Patients with EEG alterations**	EEG reported n=49 (21.5%)<br>• 28 normal (57.1%)<br>• 12 epileptiform (24.5%)<br>• 8 slowing (16.3%)<br>• 1 prolonged evoked potentials (2.0%)	EEG reported n=7 (12.1%)<br>• 4 normal (57.1%)<br>• 2 slowing (28.6%)<br>• 1 epileptiform (14.3%)	EEG reported n=6 (10.9%)<br>• 4 normal (66.7%)<br>• 2 epileptiform (33.3%)	EEG reported n=11 (39.3%)<br>• 7 normal (63.6%)<br>• 3 slowing (27.3%)<br>• 1 epileptiform (9.1%)	EEG reported n=11 (40.7%)<br>• 7 normal (63.6%)<br>• 2 slowing (18.6%)<br>• 1 epileptiform (9.1%)<br>• 1 prolonged evoked potentials (9.1%)	EEG reported n=2 (10.5%)<br>• 1 normal (50.0%)<br>• 1 epileptiform (50.0%)	EEG reported n=2 (11.8%)<br>• 2 normal (100.0%)	EEG reported n=7 (63.6%)<br>• 6 epileptiform (85.7%)<br>• 1 slowing (14.3%)	EEG reported n=2 (22.2%)<br>• 2 normal (100.0%)	EEG reported n=0 (0.0%)	EEG reported n=7 (100.0%)<br>• 1 normal (100.0%)
**Patients with MRI/CT alterations**	MRI/ CT reported n=179 (78.5%)<br>• 48 atrophy/ degeneration (26.8%)<br>• 35 infarction/ ischemic/ parenchymal defect (19.6%)<br>• 28 normal (15.6%)<br>• 24 patients with multiple alterations (13.4%)<br>• 22 white matter lesions (12.3%)<br>• 19 tumor (10.1%)<br>• 12 (post) inflammatory lesions (6.7%)<br>• 11 calcification/ sclerosis (6.1%)<br>• 7 grey matter lesions (3.9%)<br>• 7 hemorrhage (3.9%)<br>• 5 other/ unusual (2.8%)<br>• 4 cysts (2.2%)<br>• 3 eye of the tiger sign (1.7%)<br>• 2 hydrocephalus (1.1%)	MRI/ CT reported n=54 (93.1%)<br>• 26 infarction/ ischemic/ parenchymal defect (48.1%)<br>• 12 atrophy/ degeneration (22.2%)<br>• 10 patients with multiple alterations (18.5%)<br>• 7 tumor (13.0%)<br>• 4 cysts (7.4%)<br>• 4 white matter lesions (7.4%)<br>• 2 grey matter lesions (3.7%)<br>• 2 hemorrhage (3.7%)<br>• 2 calcification/ sclerosis (3.7%)<br>• 2 hydrocephalus (3.7%)<br>• 1 (post) inflammatory lesion (1.9%)<br>• 1 normal (1.9%)	MRI/ CT reported n=31 (56.4%)<br>• 9 atrophy/ degeneration (29.0%)<br>• 8 calcification/ sclerosis (25.8%)<br>• 4 normal (12.9%)<br>• 4 white matter lesions (12.9%)<br>• 3 eye of the tiger sign (9.7%)<br>• 1 infarction/ ischemic/ parenchymal defect (3.2%)<br>• 1 anatomical variant (3.2%)<br>• 1 tumor (3.2%)<br>• 1 patient with multiple alterations (3.2%)<br>1	MRI/ CT reported n=24 (85.7%)<br>• 9 normal (37.5%)<br>• 5 white matter lesions (20.8%)<br>• 5 patients with multiple alterations (20.8%)<br>• 4 hemorrhage (16.7%)<br>• 4 atrophy/ degeneration (16.7%)<br>• 3 infarction/ ischemic/ parenchymal defect (12.5%)<br>• 3 grey matter lesions (12.5%)<br>• 1 other/ unusual (4.2%)	MRI/ CT reported n=25 (92.6%)<br>• 9 (post) inflammatory lesions (36.0%)<br>• 6 normal (24.0%)<br>• 5 white matter lesions (20.0%)<br>• 5 atrophy/ degeneration (20.0%)<br>• 3 patient with multiple alterations (12.0%)<br>• 2 infarction/ ischemic/ parenchymal defect (8.0%)<br>• 1 tumor (4.0%)	MRI/ CT reported n=14 (73.7%)<br>• 9 tumor (64.3%)<br>• 3 white matter lesions (21.4%)<br>• 2 patients with multiple alterations (14.3%)<br>• 1 normal (7.1%)<br>• 1 atrophy/ degeneration (7.1%)<br>• 1 grey matter lesions (7.1%)	MRI/ CT reported n=16 (94.1%)<br>• 15 atrophy/ degeneration (93.8%)<br>• 3 patients with multiple alterations (18.6%)<br>• 2 infarction/ ischemic/ parenchymal defect (12.5%)<br>• 1 calcification/ sclerosis (6.3%)<br>• 1 hemorrhage (6.3%)	MRI/ CT reported n=6 (54.5%)<br>• 4 normal (66.7%)<br>• 1 atrophy/ degeneration (16.7%)<br>• 1 grey matter lesions (16.7%)	MRI/ CT reported n=7 (77.8%)<br>• 2 (post)-inflammatory lesions (28.6%)<br>• 1 normal (14.3%)<br>• 1 atrophy/ degeneration (14.3%)<br>• 1 infarction/ ischemic/ parenchymal defect (14.3%)<br>• 1 white matter lesions (14.3%)	MRI/ CT reported n=1 (33.3%)<br>• 1 normal (100.0%)	MRI/ CT reported n=1 (100%)<br>• 1 normal (100%)

	• 1 anatomical variant (0.6%)										
**Patients with CSF alterations**	CSF reported n=27 (11.8%)<br><br>• 16 normal (59.3%)<br>• 7 inflammatory (25.9%)<br>• 2 BBB dysfunction (7.4%)<br>• 2 elevated lactate (7.4%)	CSF reported n=2 (3.4%)<br><br>2 normal (100.0%)	CSF reported n=1 (1.8%)<br><br>1 elevated lactate (100%)	CSF reported n=0 (0%)	CSF reported n=13 (48.1%)<br><br>• 7 inflammatory (53.8%)<br>• 4 normal (30.8%)<br>• 1 BBB dysfunction (7.7%)<br>• 1 elevated lactate (7.7%)	CSF reported n=1 (5.3%)<br><br>1 normal (100%)	CSF reported n=4 (23.5%)<br><br>• 3 normal (75.0%)<br>• 1 BBB dysfunction (25.0%)	CSF reported n=1 (9.1%)<br><br>1 normal (100.0%)	CSF reported n=4 (44.0%)<br><br>• 4 normal (100.0%)	CSF reported n=0 (0.0%)	CSF reported n= 1 (100%)<br><br>1 normal (100.0%)
**Patients with SPECT/PET alterations**	SPECT/PET reported n=11 (4.8%)<br><br>• 7 hypometabolism (63.6%)<br>• 3 normal (27.3%)<br>• 1 hypermetabolism (9.1%)	SPECT/PET reported n=0 (0%)	SPECT/PET reported n=0 (0%)	SPECT/PET reported n=0 (0%)	SPECT/PET reported n=3 (11.1%)<br><br>• 2 normal (66.7%)<br>• 1 hypometabolism (33.3%)	SPECT/PET reported n=2 (10.5%)<br><br>• 1 normal (50.0%)<br>• 1 hypermetabolism (50.0%)	SPECT/PET reported n= 6 (35.3%)<br><br>• 6 hypometabolism (100%)	SPECT/PET reported n=0 (0%)	SPECT/PET reported n=0 (0%)	SPECT/PET reported n=0 (0%)	SPECT/PET reported n=0 (0%)
**Region of brain involve-ment**	Reported n=102 (44.7%)<br><br>• 35 frontal lobe (34.3%)<br>• 28 patients with multiple brain regions involved (27.5%)<br>• 27 basal ganglia (26.5%)<br>• 22 temporal lobe (21.6%)<br>• 10 parietal lobe (9.8%)<br>• 8 cerebellum (7.8%)<br>• 6 hippocampus (5.9%)<br>• 5 occipital lobe (4.9%)<br>• 4 thalamus (3.9%)<br>• 3 infarction of middle cerebral artery (2.9%)<br>• 2 associative cortex (2.0%)<br>• 2 pineal gland (2.0%)<br>• 2 midbrain (2.0%)<br>• 2 pons (2.0%)<br>• 1 insula (1.0%)<br>• 1 acoustic nerve (1.0%)<br>• 1 posteromedial cortex (1.0%)<br>• 1 substantia nigra (1.0%)<br>• 1 visual cortex (1.0%)<br>• 1 suprasellar cistern (1.0%)<br>• 1 spinal cord (1.0%)<br>• 1 corona radiata (1.0%)<br>• 1 superior sagittal sinus (1.0%)<br>• 1 internal capsule (1.0%)	Reported n=48 (82.8%)<br><br>• 19 basal ganglia (39.6%)<br>• 15 frontal lobe (31.3%)<br>• 13 patients with multiple brain regions involved (27.1%)<br>• 7 parietal lobe (14.6%)<br>• 6 cerebellum (12.5%)<br>• 5 temporal lobe (10.4%)<br>• 4 thalamus (8.3%)<br>• 2 infarction of middle cerebral artery (4.2%)<br>• 1 hippocampus (2.1%)<br>• 1 occipital lobe (2.1%)<br>• 1 corona radiata (2.1%)<br>• 1 superior sagittal sinus (2.1%)<br>• 1 internal capsule (2.1%)<br>• 1 pons (2.1%)<br>• 1 paraventricular (2.1%)	Reported n=2 (3.6%)<br><br>• 1 frontal lobe (50.0%)<br>• 1 parietal lobe (50.0%)<br>• 1 patient with multiple brain regions involved (50.0%)<br>• 1 paraventricular (50.0%)	Reported n=14 (50.0%)<br><br>• 12 frontal lobe (85.7%)<br>• 7 patients with multiple brain regions involved (50.0%)<br>• 7 temporal lobe (50.0%)<br>• 2 occipital lobe (14.2%)<br>• 1 associative cortex (7.1%)<br>• 1 substantia nigra (7.1%)<br>• 1 parietal lobe (7.1%)	Reported n=4 (14.8%)<br><br>• 2 basal ganglia (50.0%)<br>• 2 temporal lobe (50.0%)<br>• 1 patient with multiple brain regions involved (25.0%)<br>• 1 hippocampus (25.0 %)	Reported n=15 (78.9%)<br><br>• 3 patients with multiple brain regions involved (20.0%)<br>• 2 temporal lobe (13.3%)<br>• 2 basal ganglia (13.3%)<br>• 2 pineal gland (13.3%)<br>• 2 midbrain (13.3%)<br>• 1 hippocampus (6.7%)<br>• 1 frontal lobe (6.7%)<br>• 1 parietal lobe (6.7%)<br>• 1 infarction of middle cerebral artery (6.7%)<br>• 1 insula (6.7%)<br>• 1 acoustic nerve (6.7%)<br>• 1 cerebellum (6.7%)<br>• 1 pons (6.7%)<br>• 1 suprasellar cistern (6.7%)<br>• 1 spinal cord (6.7%)	Reported n=9 (52.9%)<br><br>• 4 frontal lobe (44.4%)<br>• 4 basal ganglia (44.4%)<br>• 3 patients with multiple brain regions involved (33.3%)<br>• 3 temporal lobe (33.3%)<br>• 1 posteromedial cortex (11.1%)<br>• 1 occipital lobe (11.1%)<br>• 1 associative cortex (11.1%)<br>• 1 visual cortex (11.1%)	Reported n=9 (81.8%)<br><br>• 3 temporal lobe (33.3%)<br>• 3 hippocampus (33.3%)<br>• 2 frontal lobe (22.2%)<br>• 1 occipital lobe (11.1%)	Reported n=0 (44.4%)	Reported n=0 (33.3%)	Reported n=1 (100.0%)<br><br>• 1 cerebellum (100%)
**Therapy response**	**Therapy reported n=181 (79.4%)**<br><br>Successful therapy n=129 (71.3%) with<br><br>• 30 only conventional: SSRI (16.6%)<br>• 21 only causal therapy (11.6%)<br>• 21 non-conventional psychopharmaceutic + SSRI (11.6%)<br>• 8 causal therapy + SSRI (4.4%)<br>• 8 only non-conventional psychopharmaceutic (4.4%)<br>• 9 only conventional: SSRI + CBT (5.0%)	**Therapy reported n=49 (84.5%)**<br><br>Successful therapy n=35 (71.4%) with<br><br>• 13 only conventional: SSRI (26.5%)<br>• 5 non-conventional psycho-pharmaceutic + SSRI (10.2%)<br>• 3 only causal therapy (6.1%)<br>• 2 only conventional: CBT (4.1%)<br>• 2 only non-conventional psychopharmaceutic (4.1%)<br>• 2 only lithium (4.1%)<br>• 1 only conventional: SSRI + CBT (2.0%)	**Therapy reported n=36 (65.5%)**<br><br>Successful therapy n=28 (77.8%) with<br><br>• 10 only conventional: SSRI (27.8%)<br>• 7 non-conventional psycho-pharmaceutic + SSRI (19.4)<br>• 2 causal therapy + SSRI (5.6%)<br>• 2 only DBS (5.6%)<br>• 1 only conventional: clomipramine (2.8%)<br>• 1 only conventional: SSRI + CBT (2.8%)<br>• 1 only conventional: SSRI + clomipramine + CBT (2.8%)<br>• 1 only DBS (2.8%)	**Therapy reported n=21 (75.0%)**<br><br>Successful therapy n=16 (76.2%) with<br><br>• 2 non-conventional psycho-pharmaceutic + SSRI (9.5%)<br>• 3 only conventional: SSRI + CBT (14.3%)<br>• 3 only conventional: SSRI (14.3%)<br>• 1 only conventional: SSRI + clomipramine (4.8%)<br>• 1 only non-conventional	**Therapy reported n=26 (96.3%)**<br><br>Successful therapy n=18 (69.2%) with<br><br>• 8 only causal therapy (30.8%)<br>• 2 causal therapy + SSRI (7.7%)<br>• 2 only conventional: SSRI (7.7%)<br>• 2 only conventional: SSRI + CBT (7.7%)<br>• 1 non-conventional psychopharmaceutic + SSRI (3.8%)<br>• 1 non-conventional psychopharmaceutic + clomipramine (3.8%)<br>• 1 non-conventional psycho-pharmaceutic +	**Therapy reported n=13 (68.4%)**<br><br>Successful therapy n=7 (53.8%) with<br><br>• 4 non-conventional psycho-pharmaceutic + SSRI (30.8%)<br>• 2 only non-conventional psycho-pharmaceutic (15.4%)<br>• 1 only causal therapy (7.7%)<br><br>Unclear if therapy was successful n=1 (7.7%)<br><br>Therapy not successful n=5 (38.5%)	**Therapy reported n=13 (76.5%)**<br><br>Successful therapy n=6 (46.2%) with<br><br>• 2 non-conventional psycho-pharmaceutic + SSRI (15.4%)<br>• 1 non-conventional psychotherapy +SSRI (7.7%)<br>• 1 only non-conventional psycho-pharmaceutic (7.7%)<br>• 1 only causal therapy (7.7%)<br>• 1 causal therapy + SSRI (7.7%)	**Therapy reported n=11 (100%)**<br><br>Successful therapy n=10 (90.9%) with<br><br>• 5 only causal therapy (45.5%)<br>• 2 only conventional: CBT (18.2%)<br>• 1 causal therapy + SSRI (9.1%)<br>• 1 causal therapy + CBT (9.1%)<br>• Non-conventional psychopharmaceutic + SSRI + clomipramine (9.1%)	**Therapy reported n=9 (100%)**<br><br>Successful therapy n=7 (77.8%) with<br><br>• 2 only conventional: SSRI (22.2%)<br>• 2 only conventional: SSRI + CBT (22.2%)<br>• 2 causal therapy + SSRI (22.2%)<br>• 1 only non-conventional psycho-pharmaceutic (11.1%)	**Therapy reported n=3 (100%)**<br><br>Successful therapy n=3 (66.7%) with<br><br>• 2 only causal therapy (66.7%)<br><br>Therapy not successful n=1 (33.3%)<br><br>**Therapy not reported n=1 (33.3%)**	**Therapy reported n=1 (100%)**<br><br>Successful therapy n=0 (0%)<br><br>Therapy not successful n=1 (100%)

	• 5 only conventional: CBT (2.8%)<br>• 4 only conventional: clomipramine (2.2%)<br>• 3 causal therapy + SSRI + CBT (1.6%)<br>• 2 causal therapy + CBT (1.1%)<br>• 2 non-conventional psychopharmaceutic + SSRI + clomipramine (1.1%)<br>• 2 only lithium (1.1%)<br>• 2 non-conventional psychotherapy + SSRI (1.1%)<br>• 2 only DBS (1.1%)<br>• 1 only conventional: SSRI + clomipramine + CBT (0.6%)<br>• 1 only conventional: SSRI + clomipramine (0.6%)<br>• 1 only conventional: clomipramine + CBT (0.6%)<br>• 1 non-conventional psychopharmaceutic + clomipramine (0.6%)<br>• 1 non-conventional psychopharmaceutic + SSRI + CBT (0.6%)<br>• 1 only non-conventional psychotherapy (0.6%)<br>• 1 specific cause of improvement unclear (0.6%)<br>• 1 causal therapy + SSRI + clomipramine (0.6%)<br>• 1 only cannabinoid (0.6%)<br>• 1 only ECT (0.6%)<br>• 1 ECT + SSRI (0.6%)<br>Unclear if therapy was successful n=11 (6.1%)<br><br>Therapy not successful n=41 (21.7%)<br><br>**Therapy not reported n=45 (19.7%)**	• 1 only conventional: clomipramine + CBT (2.0%)<br>• 1 non-conventional psycho-pharmaceutic + SSRI + CBT (2.0%)<br>• 1 non-conventional psychotherapy + SSRI (2.0%)<br>• 1 causal therapy + SSRI + CBT (2.0%)<br>• 1 only conventional: clomipramine (2.0%)<br>• 1 causal therapy + SSRI + clomipramine (2.0%)<br>• 1 only cannabinoid (2.0%)<br><br>Unclear if therapy was successful n=4 (8.2%)<br><br>Therapy not successful n=10 (20.4%)<br><br>**Therapy not reported n=9 (15.5%)**	• 1 only non-conventional psycho-pharmaceutic (2.8%)<br>• 1 causal therapy + CBT (2.8%)<br>• 1 causal therapy + SSRI + CBT (2.8%)<br>• 1 ECT + SSRI (2.8%)<br><br>Unclear if therapy was successful n=3 (8.3%)<br><br>Therapy not successful n=5 (13.9%)<br><br>**Therapy not reported n=19 (34.5%)**	psychopharmaceutic (4.8%)<br>• 1 only non-conventional psychotherapy (4.8%)<br>• 1 only causal therapy (4.8%)<br>• 1 only ECT (4.8%)<br><br>Therapy not successful n=5 (23.8%)<br><br>**Therapy not reported n=6 (21.4%)**	SSRI + clomipramine (3.8%)<br>• 1 specific cause of improvement unclear (3.8%)<br><br>Unclear if therapy was successful n=1 (3.8%)<br><br>Therapy not successful n=7 (26.9%)<br><br>**Therapy not reported n=1 (3.7%)**	**Therapy not reported n=6 (31.6%)**	Unclear if therapy was successful n=1 (7.7%)<br><br>Therapy not successful n=6 (46.2%)<br><br>**Therapy not reported n=4 (23.5%)**	Therapy not successful n=1 (9.1%)	Unclear if therapy was successful n=1 (11.1%)<br><br>Therapy not successful n=1 (11.1%)		

Supplementary Table 1: Complete findings of all cases with suspected secondary obsessive-compulsive syndromes. Abbreviations: CBT, Cognitive-behavioral therapy; CNS, Central nervous system; CSF, Cerebrospinal fluid; CT, Computer tomography; DBT, Dialectical behavior therapy; ECT, electroconvulsive therapy; EEG, Electroencephalography; MELAS, mitochondrial disease; MRI, magnetic resonance imaging; OCD, Obsessive compulsive disorder; OCS, Obsessive compulsive symptoms; PCDH19, Procadherin-19; PET, Positron-emission-tomography; SD, standard deviation; SHANK2, SH3 and Multiple Ankyrin Repeat Domains 2; SPECT, Single-Photon-Emission-Computer tomography; SSRI, selective serotonin reuptake inhibitor; Y-BOCS, Yale-Brown Compulsive Obsessive Scale.
